# Recombination Hotspots and Population Structure in *Plasmodium falciparum*

**DOI:** 10.1371/journal.pbio.0030335

**Published:** 2005-09-13

**Authors:** Jianbing Mu, Philip Awadalla, Junhui Duan, Kate M McGee, Deirdre A Joy, Gilean A. T McVean, Xin-zhuan Su

**Affiliations:** **1** Laboratory of Malaria and Vector Research, National Institutes of Health, Rockville, Maryland, United States of America,; **2** Department of Genetics, North Carolina State University, Raleigh, North Carolina, United States of America,; **3** Department of Statistics, University of Oxford, Oxford, United Kingdom; Rutgers UniversityUnited States of America

## Abstract

Understanding the influences of population structure, selection, and recombination on polymorphism and linkage disequilibrium (LD) is integral to mapping genes contributing to drug resistance or virulence in *Plasmodium falciparum*. The parasite's short generation time, coupled with a high cross-over rate, can cause rapid LD break-down. However, observations of low genetic variation have led to suggestions of effective clonality: selfing, population admixture, and selection may preserve LD in populations. Indeed, extensive LD surrounding drug-resistant genes has been observed, indicating that recombination and selection play important roles in shaping recent parasite genome evolution. These studies, however, provide only limited information about haplotype variation at local scales. Here we describe the first (to our knowledge) chromosome-wide SNP haplotype and population recombination maps for a global collection of malaria parasites, including the 3D7 isolate, whose genome has been sequenced previously. The parasites are clustered according to continental origin, but alternative groupings were obtained using SNPs at 37 putative transporter genes that are potentially under selection. Geographic isolation and highly variable multiple infection rates are the major factors affecting haplotype structure. Variation in effective recombination rates is high, both among populations and along the chromosome, with recombination hotspots conserved among populations at chromosome ends. This study supports the feasibility of genome-wide association studies in some parasite populations.

## Introduction

The interaction between *Plasmodium falciparum* and humans has been a potent selective force in the evolution of both species. The high mortality rate—an estimated 1.1–2.7 million people die each year from malaria [1]—leads to strong selection, both on host genes that contribute to resistance [2,3], and on parasite genes involved in the infection process [4]. Colonization of novel populations and the use of anti-malarial drugs have imposed additional selective forces on the parasite genome [5–7]. The signatures of selection can potentially be used to map genes associated with drug resistance, local adaptation, or antigenic interactions [8]. Two key factors, however, limit the power of population-based approaches to gene mapping. First, population structure, caused either by geographic isolation or epidemiologic stratification, can generate spurious association between phenotypic and genetic variation. Second, the extent of linkage disequilibrium (LD) within populations, which determines both the number of markers required for association studies and the mapping resolution, is unknown. LD can be influenced by diverse factors, including the recombination rate, the local parasite effective population size, population differentiation, and the extent of inbreeding [9]. Determining the extent to which geography, recombination, and host-parasite interactions have shaped genomic structure requires analysis of global-scale patterns of genetic variation.

For *P. falciparum*, data describing population structure and LD have come mostly from nucleotide polymorphisms in a number of genes encoding candidate malaria vaccines that may be under strong host immune selection [10–12]. More “neutral” microsatellite markers sampled from across the genome have also been employed to study *P. falciparum* populations. In a study using 12 microsatellites, parasite populations were clustered into major groups based on their continental origins, with the majority of variation found within locations in Africa and Papua New Guinea (PNG), but also between subpopulations from different sampling sites in South America (Brazil, Colombia, and Bolivia) [13]. Because the microsatellites were located on different chromosomes, the study could not provide information about the chromosomal scale of LD and variation in population recombination rates. Similarly, significant microsatellite allele-sharing was found among South American isolates, but not among those from Africa, suggesting distinct population structure in different continents [5]. Although variation in cross-over rates was observed in a meiotic map constructed for *P. falciparum* [14,15], the power to detect recombination variation using genetic crosses is limited. In contrast, population genetic data provide greater resolution in detecting historical recombination events, which can be estimated from events occurring over generations ago.

In both the yeast and human genomes, recombination principally occurs at specific regions called “hotspots” [16–19]. However, very limited information on recombination hotspot locations at a genome-wide scale is available in other organisms, particularly for important human pathogens such as *P. falciparum*. The molecular mechanisms determining hotspot location and activity are largely unknown. In yeast, chromatin structure influences the initiation of double-strand breaks at hotspots [18], but no specific sequence or motif has been identified as causing recombination hotspots. The observation of meiotic drive associated with hotspots has led some to suggest that they may be short-lived because of selection against sites that initiate double-strand breaks [20]. Identification of recombination hotspots and a better understanding of the mechanism underlying hotspot activity will not only facilitate association studies, but also shed light on *P. falciparum* genome evolution.

Recombination allows sites to evolve independently, and thus it may act as a diversifying force, generating new variants for the parasite to evade host immunity. The malaria parasite has asexual and sexual stages in its life history. In natural *P. falciparum* populations, recombination events will be observed only when a host is infected with multiple genotypes. As a result, “effective” population recombination rates are directly correlated with frequencies of multiple infections, endemicity, and the out-crossing rate [21]. Understanding the patterns of variation in recombination rates along the chromosome is critical for detecting regions that have been potentially subjected to selective sweeps [22]. To study the nature and scale of LD and recombination variation in the parasite genome, we assayed 99 worldwide *P. falciparum* isolates (Figure S1) and one chimpanzee parasite *(P. reichenowi)* for single nucleotide polymorphisms (SNPs) spanning Chromosome 3, at an average interval of one SNP per ∼5.5 kilobases (kb) [15]. We show high variation in recombination rates, among different parasite populations, and along the length of Chromosome 3. The presence of LD in some populations, and selection by anti-malarial agents, support the possibility of genome-wide association for genes affecting drug response of malaria parasites.

## Results/Discussion

### SNP Collection and Distribution

The SNPs were ascertained by re-sequencing based on variant sites identified from a panel of five global isolates [15]. Of the 183 SNPs that produced genotypes for most of the 99 isolates, 138 (75.4%) are SNPs with a global minor allele frequency greater than or equal to 0.02. Although 105 SNPs segregate in more than one of the four major regions surveyed—Africa, Southeast (SE) Asia, South and Central America, and PNG—only 39 segregate in all populations; 21 SNPs segregate in Africa alone, 27 in America, 16 in SE Asia, and 9 in PNG. Average pair-wise nucleotide diversity for the four major geographic regions revealed similar per-site heterozygosity values (relative values to Africa equal 1; America, 1.13; SE Asia, 0.87; and PNG, 0.91). The continent-specific SNPs suggest potential population structure in this worldwide parasite collection.

### Parasite Populations Genetically Clustered According to Their Continental Origins

**Figure 1:**
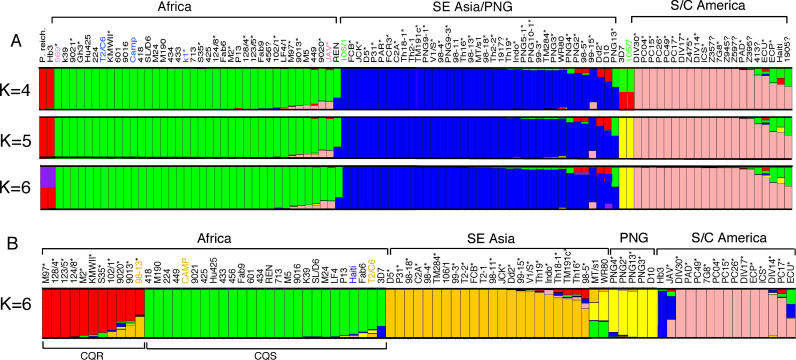
Inferred Population Structure of Global Parasite Isolates Each vertical bar represents an individual parasite with its names given at the top of the panels. Predefined numbers of populations (K = 2–8) were run ten times each, using Structure 2. (A) Population partitions using SNPs from Chromosome 3 (K = 4–6, linkage model). At K = 5, the most consistent membership coefficients were obtained. (B) Population partition using SNPs from 37 putative transporters (admixture model). Similar clustering was obtained with that of Chromosome 3, including Camp and T2/C6 (no data for K1) with African parasites and 106/1 with SE Asian parasites. However, African parasites were partitioned into chloroquine-resistant and -susceptible parasites. Note: Only 81 are available for the transporter data. *, parasites resistant to chloroquine; ?, parasites from which drug data are not available.

Because our parasites were collected from various locations at different times, population differentiation may affect our inferences of population parameters [23]. We therefore estimated potential population structure using a Bayesian model-based clustering algorithm in Structure 2 [24]. The program assumes a model with K populations, with each population characterized by a set of allele frequencies at each locus. The likelihood of a series of K values is calculated and a best K value, based on likelihood tests, is selected after a series of runs with different K values. Genotype data can be analyzed using different ancestry models, such as an admixture model for individuals with mixed ancestry, or a linkage model for recently mixed populations with linked markers. For the worldwide parasite populations, both admixture and linkage models produced similar results, with the most positive likelihood values (or best statistical support) when the number of populations (K), a priori, was assumed to be five (Figure 1A). Most individuals clustered according to continent, regardless of their location or time of isolation, with the exception of a few isolates (569, T2/C6, Camp, K1, JAV, and 106/1) possibly due to either contamination during in vitro culture or human migration. Although parasites from SE Asia and PNG grouped together, some differences can be seen (Figure 1A). The 3D7 parasite (widely believed to be from Africa) clustered with 105/7 from Sudan, differing by only four SNPs, and yet separately from the rest of the African isolates. This result suggests that the two parasites are closely related, but that the origin of 3D7 requires further investigation. Similarly, parasite isolate 106/1 is closely related to the SE Asian parasite FCB, as the two have identical chromosomal haplotypes (with the exception of one SNP), similar to results from microsatellite genotypes [5]. Parasites from Central America (HB3 and Haiti) and coastal South America (ECU and JAV) showed some differences from those of Brazil and Peru. The clustering of JAV with African parasites, despite having part of its genome shared with those of South American parasites, suggests that the parasite might have migrated from Africa more recently, and recombination had allowed its genome to be mixed with those of South American parasites. The clustering of HB3 and *P. reichenowi* may simply reflect their relatively unique genetic backgrounds, as compared with the rest of the isolates.

The continental genetic partitions were supported by analyses of population variance *F_ST_,* a fixation index describing genetic variation among populations [25] (Table 1), confirming significant population differentiation among parasites from different continents, which accounts for 24% of the total genetic variation. Although Structure 2 did not resolve differences between isolates from SE Asia and PNG, significant heterogeneity between isolates was observed when these populations were treated as separate, a priori, and hence, as individual populations, in the recombination analyses.

**Table 1:**
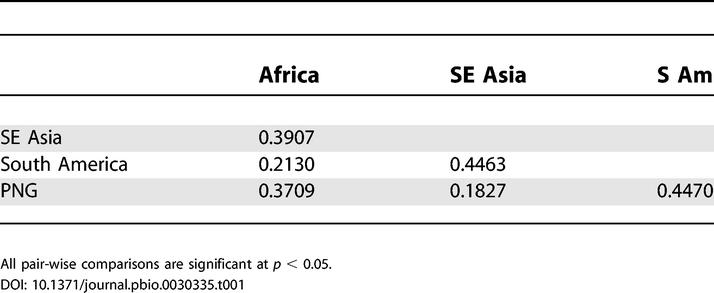
Pairwise *F_ST_* of Subpopulations from Different Regions of the World

### Effects of Drug Selection on the Inference of Population Structure

Chromosome-wide variants provide not only a means for inferring population structure, but also a genomic control for comparison with other variants that are functionally significant, with respect to evasion of host immunity or drug resistance. We compared the inferred population structure from Chromosome 3 variants with estimates using 102 SNPs from 37 putative transporter genes on different chromosomes (Table S1), some of which were shown to be associated with chloroquine resistance [26]. The membership partition of parasite isolates using the transporter SNPs is largely concordant with those from Chromosome 3 variants, grouping the parasites into major geographic boundaries; however, obvious exceptions are the presence of two clear subpopulations in Africa, and the separation of PNG parasites from SE Asian parasites (Figure 1B). Interestingly, the subpopulations in Africa are partitioned according to parasite responses to chloroquine, i.e., one group consisting of chloroquine-resistant parasites (red bars in Figure 1B) and the other of chloroquine-sensitive parasites, regardless of sampling location within Africa, including parasites simultaneously isolated from one location, such as those from Ghana (9013, 9016, 9020, 9021). In contrast, clustering the African parasites using Chromosome 3 SNPs at K = 2 did not separate the African parasites into resistant and sensitive groups (unpublished data). Similarly, the separation of PNG isolates from SE Asian parasites could be due to the independent origin of chloroquine-resistance founder mutations in PNG. These results illustrate how selection on specific genes can greatly influence estimates of population structures.

### High Recombination Rate Variation among Different Parasite Populations

A key factor in the success of association studies is the detection of variation in LD and population recombination rates, within and between populations. Here we examined the evidence for variable recombination rates, among populations and along chromosomes, using recently developed parametric [27] and nonparametric [28] methods (see Materials and Methods), respectively. The parametric methods use a model to infer the effective population recombination rate [2*N_e_r*(1 − *f*)], where *N_e_* is the effective population size, *r* is the per-generation recombination rate, and *f* is the inbreeding coefficient; the nonparametric method estimates the minimum number of recombination events *(Rh).* We cannot separate the effects of *N_e_, r,* and *f* on individual estimate of a sample, but we can show the relationship of *f* on estimates of the effective population recombination parameter nonetheless. This is because the variation in estimates of recombination rates among populations is due to variation in transmission frequencies and/or *f*. The parametric methods have been shown to be robust in the context of alternative mutation models and have high power to detect recombination, even with SNP ascertainment bias [19,27].

Chromosome-wide estimates of the population recombination rate parameter ranged from ∼400 per Mb in the American to over 10^5^ per Mb in the African populations (Table 2). Although the Structure 2 analyses did not point to a distinction between PNG and SE Asia populations, *F_ST_* estimates suggest significant differentiation between the two. We therefore calculated the rates for PNG (*n* = 11) alone and in combination with SE Asia (*n* = 29, combined *n* = 40). The PNG population has the smallest *Rh* estimate but a large population recombination rate estimate; however, the rate estimate has a large confidence interval, due in part to the small sample size. When the PNG and SE Asian populations were combined, estimates of the effective population recombination rate (1,667) were more similar to SE Asia estimates alone (Table 2) with narrower confidence intervals (877–1,916). These estimates point to extreme differences in the effective population recombination rates, which can result from differences in transmission rates, inbreeding, and effective population sizes, an observation that is itself unparalleled.

**Table 2:**
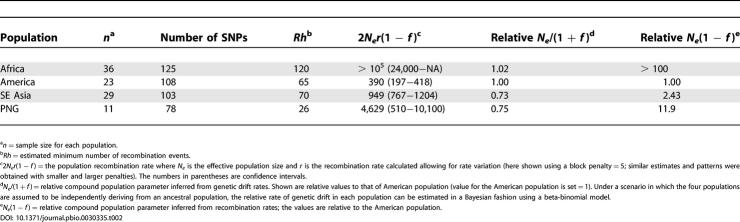
Population Recombination Rate Variation on Chromosome 3 among *P. falciparum* Populations

In addition to measuring differences in recombination events among populations, the chromosome-wide data are also informative for evaluating the influence of genetic drift and recombination on genetic variation. In partially out-crossing species, population genetic parameters can be scaled by the inbreeding coefficients, *f* [21,29]. In *P. falciparum,* inbreeding coefficients have been shown to be directly correlated with rates of transmission and/or frequencies of multiple infection [30]. Here we compared relative values of drift, *N_e_*/(1 + *f*) (see Materials and Methods), estimated from nucleotide variation in a Bayesian fashion, through the use of a beta-binomial model [23,31], and *N_e_*(1 *− f*) from our population recombination rate estimates (Table 2). The beta-binomial model measures the difference of each population from a hypothetical average, or ancestral, population by a parameter, *c_j_*, for each population, *j* [31]. These parameters can be thought of as a generalization of *F_ST_* [23]. We can show relative estimates (to the American population) only, not the actual estimates of *N_e_,* as inferences would be affected by SNP ascertainment bias in an unknown manner. All populations are similar in terms of relative rates of drift. For example, SE Asia and PNG parasites have similar population mutation rates and therefore similar rates of genetic drift, but the SE Asian population has a significantly reduced recombination rate relative to the African population, indicating a higher rate of inbreeding. Although the African population has a comparable rate of genetic drift with that of American isolates, it has a recombination rate at least two orders of magnitude larger. These results show that differences in transmission frequencies and rates of multiple infections among populations, rather than effective population sizes, are most important in shaping haplotype variation among populations and in determining local levels of LD.

### Recombination Hotspots Conserved at the Middle and at the Ends of the Chromosome

**Figure 2:**
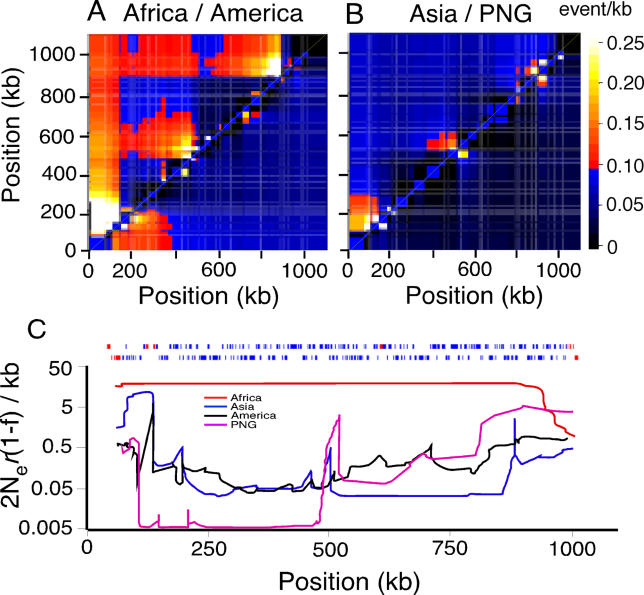
The Distribution of Detectable Recombination Events on Chromosome 3 of *P. falciparum* In (A) and (B), each panel shows, for two populations, a minimum number of recombination events (assuming an infinite-sites model) between each pair of segregating sites, scaled by physical distance to identify regions of high and low recombination. (A) African (upper) and American (lower) populations. (B) SE Asian (upper) and PNG (lower) populations. The color bar unit is recombination event/kb. (C) Estimates of population recombination rate variation for African (red line), SE Asian (blue), American (black), and PNG (purple) samples using the RJMCMC method with a jump penalty of five. Shown on top are the locations of genes on the plus (top) and minus (below) strands, with known cell-surface genes in red [34].

We next examined whether recombination rates were uniform along the chromosome, because variation in recombination rates will dramatically affect the effectiveness of association studies [32]. The majority of recombination events cluster near the chromosome ends and in the middle of the chromosome (Figure 2A and 2B). Nonparametric estimates revealed many recombination events, as well as recombination hotspots, among African parasites (Figure 2A). Similar recombination hotspots were also found in the remaining parasite populations, except American, where the hotspot in the middle of the chromosome is absent. Parametric methods, based on coalescent models, also detected significant recombination rate variation (SE Asian, *p* < 0.001; American, *p* < 0.001; PNG, *p* < 0.05) in all populations except African, for which the high levels of historic recombination invalidated the test. Figure 2C shows the recombination map along the chromosome for the four populations, as estimated by the Reversible Jump Markov Chain Monte Carlo (RJMCMC) method [19]. Even though the sample size for PNG is relatively small for these types of rate estimates [27,33] and the confidence intervals for the overall PNG rate is large, the inferred location of recombination hotspots using the two approaches generally concur (Table 2).

Although the overall population recombination rate is highly variable among populations, the chromosomal locations of major recombination hotspots were conserved. Subtelomeric regions in *P. falciparum* clearly exhibit elevated crossing over, similar to those observed in human males (but not females) [32]. The conservation is likely due, in part, to the shared evolutionary ancestry of *P. falciparum* populations. Additionally, these regions contain a high density of genes such as *var, rifin,* and *stevor,* whose products are implicated in cell-surface interactions [34] and are consequently under strong immune-mediated diversifying selection (as demonstrated by the high rate of amino acid evolution) [35]. Increases in recombination can be indirectly selected, either because recombination generates genetic variation at these genes or because it allows sites that are targeted by selection to freely evolve without interfering with each other [36]. Alternatively, these gene families themselves may contribute to increased recombination through concerted evolutionary processes, such as unequal rates of cross-over. Regardless, these observations suggest that elevated recombination rates may play a significant role in generating multiple haplotypes at genes important for *P. falciparum's* evasion of host immunity.

### Variation in LD and Haplotype Blocks among Parasite Populations

**Figure 3:**
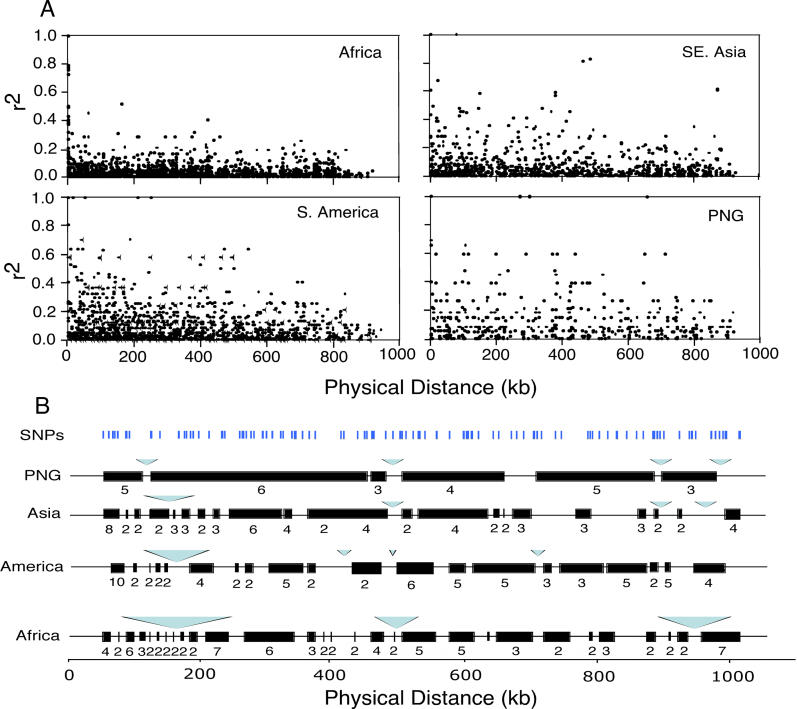
LD and Haplotype Blocks across Chromosome 3 of *P. falciparum* (A) LD decays with increasing distances between variable sites along Chromosome 3 in populations from Africa, SE Asia, S America, and PNG. The LD index r^2^ (square of correlation coefficient) were calculated considering all pair-wise values for SNPs with minor allele frequency > 5%. Parasite isolates K1, Camp, T2/C6, 105/7, 3D7, HB3, Haiti, JAV, ECP, 569, and 1905 are excluded from the LD analyses because these parasites were placed in clusters different from locations they were isolated from or have different genetic backgrounds. (B) Haplotype blocks defined as regions where all pairs of sites have *D*′ ≥ 0.8. Values below each block are the number of htSNPs required to capture all haplotype variants when haplotype blocks are characterized by complete association among variants (r^2^ = 1). Bars on the top are locations of assayed SNPs. Triangles denote a site in the middle of a recombination hotspot, and the width of the triangles represents the region hotspot spans.

Population genetic models predict that the extent of LD is inversely proportional to the population recombination rate [37,38]. As expected, we observed less LD in African populations. Indeed, LD decays with increasing physical distance between pairs of segregating sites in all populations, but more slowly in SE Asian and American populations relative to parasites from Africa, where LD decays rapidly over very short distances (Figure 3A).

Critical for association studies is the identification of haplotype blocks and the minimal set of haplotype tagging SNPs (htSNPs) required to capture haplotype variation in a population sufficiently, which will reduce cost and effort. Haplotype blocks of various sizes were identified for the four populations (Figure 3B). The African population, with its high inferred population recombination rate, clearly had the smallest average block length (11.2 kb) and the greatest number of blocks (*n* = 46), whereas the average block size for PNG was 56 kb, with only 11 blocks defined. Again, the relatively large blocks in PNG could be due to a small sample size and/or sampling from a small isolated area or population. Relatively high inbreeding frequency (0.915) [12] could also contribute to the large haplotype blocks. Among African populations, the number of htSNPs required to capture haplotype variation sufficiently was 53% of the SNPs polymorphic in African parasites. Whereas major hotspots are clearly breaking up block structure in all populations near the chromosome ends (Figure 2B), other recombination events are also disrupting LD and haplotype blocks (Figures 2 and 3) in different regions.

A primary objective in studying population recombination rate variation and haplotype maps in *P. falciparum* is to facilitate identification of genes responsible for important parasite traits, such as drug resistance and virulence. There is currently tremendous interest in using LD to map human disease genes. The present results suggest that LD mapping may, in some circumstances, be more effective in studying partially selfing species such as *P. falciparum* than out-crossing species, such as humans or *Drosophila,* because LD can persist over extensive genomic regions. The overall estimated population recombination rates clearly vary among populations, primarily due to different inbreeding and transmission rates, but the chromosomal locations of major hotspots are conserved. Population structure among sub-Saharan African parasites appears to be minimal because of high estimated recombination rates; however, very high marker densities may be required for mapping even newly arisen traits, because traces of LD between loci will be lost quickly. The presence of chromosomal segments with low population recombination rates in SE Asian and American parasites suggests it is possible to conduct genome-wide association mapping for certain phenotypes in these geographic locations, using relatively low densities of marker loci. This approach is likely to be particularly effective for genes involved in drug resistance, since the mutations involved have occurred recently, allowing little time for LD between marker and trait loci to be broken down. However, as shown in this study, recombination rates are not uniform across the chromosome, and, therefore, the location of the genes of interest will be highly relevant. Studying these hotspots will ultimately illuminate the as yet mysterious factors that direct the location and frequency of recombination in this and other species. The presence of LD in at least some populations, the recent appearance of mutations conferring drug resistances, and the use of high-density genetic maps make it practical to conduct genome-wide association studies in this relatively small genome.

## Materials and Methods

### DNA sequences and SNP ascertainment

Predicted coding sequences of the 3D7 parasite were downloaded from PlasmoDB (http://www.plasmodb.org/). DNA sequences covering SNP sites identified from five isolates in our previous study [15] were amplified and sequenced from additional 94 isolates collected from different regions of the world (Figure S1). After exclusion of those SNPs that were difficult to amplify from all isolates due to high AT content or other technical reasons, 183 SNPs were obtained for analysis (see Figure 3B for physical locations on the chromosome). DNA sequences were trimmed and aligned using Phred/Phap (http://www.phrap.org/) and Sequencher 3.1 (Gene Codes, Ann Arbor, Michigan, United States) to identify SNPs. All potential SNPs and discrepancies were verified by visually inspecting chromatogram traces.

### Population structure analysis

Population structures were analyzed using the Structure 2 package [24]. We ran the program ten times at each K value (K = 2–8) with 50,000 burn-ins and 100,000 iterations. For the SNPs from Chromosome 3, both admixture and linkage models were used (admixture only for SNPs from the putative transporters). *F_ST_* values were calculated in Arlequin [25]. Inferences of effective population size assuming a beta-binomial distribution were estimated in a Bayesian fashion as in Marchini et al. [23] using the R package popgen (http://www.stats.ox.ac.uk/~marchini/software.html).

### Inference of population recombination rate

Nonparametric estimates of the number of recombination events *(Rh)* were calculated using the methodology of Myers and Griffiths [28]. Model-based parametric estimates of the recombination rate were calculated using the LDhat programs, pairwise and interval (http://www.stats.ox.ac.uk/~mcvean/LDhat/LDhat1.0/LDhat1.0.html). The method extends the composite likelihood approach of Hudson [33], to allow for recurrent mutation [27], and adopts a Bayesian implementation that uses a RJMCMC scheme to fit a recombination map composed of a series of intervals of constant rate [19]. Under simple models of demographic history, the key quantity in determining the extent of LD between alleles at linked loci is the product of the effective population size, *N_e_,* which is inversely related to the rate of genetic drift, the per-generation rate of recombination, *r,* and for *P. falciparum,* the rate of outcrossing, 1 − *f,* where *f* is the inbreeding coefficient [9,21]. Without an absolute estimate of the out-crossing rate and *N_e_* in a population, we can only estimate the compound parameter of the recombination rate, 2*N*_e_ *r*(1 − *f*). Such methods have also been used to develop an “effective recombination rate” for malaria parasites [39]. It has also been shown that the coalescent with partial selfing looks like the standard coalescent, but with rates of coalescence (1 + *f*) times faster [21,29]. If time is rescaled in units of 2*N*/(1 + *f*) generations, the coalescence process is identical to the standard coalescent. Similarly, ancestral recombination graphs with inbreeding have rates of coalescence that are (1 + *f*) times faster and rates of recombination that are (1 − *f*) times slower than in a completely out-crossing situation [29]. Again, if time is rescaled in units of 2*N*/(1 + *f*) generations, the process looks like the out-crossing case but with a recombination rate that is (1 − *f*)/(1 + *f*) times slower than in the out-crossing case. In the case of inbreeding, 4*Nμ* is therefore replaced by 4*Nμ/*(1 *+ f*), and 4*Nr* by 4*Nr*(1 − *f*).

The compound population recombination rate parameter can be estimated from population genetic data using coalescent methods adapted specifically to account for the possibility of recurrent or back mutation and for an AT-rich genome such as that of *P. falciparum* [27,33,40]. By estimating this parameter, we can evaluate how historical population size and out-crossing rate affect LD in populations. These approaches have been shown to be robust in evaluating population recombination even when the underlying nucleotide variation is variable along a chromosome [27] and with respect to SNP ascertainment bias [19]. Because of the non-independence introduced by the composite likelihood, the variance for RJMCMC is unknown, and we therefore report the marginal posterior mean recombination rate (Figure 2C) for each SNP interval as a point estimate while performing the statistical tests (below) to obtain confidence intervals for local recombination rate variation.

To test the hypothesis of constant recombination rate in each population, we used coalescent simulations to generate the distribution of a test statistic, T,



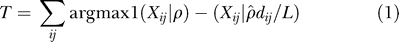



which is the sum, over all pairs of sites *X_ij_*, of the difference in log likelihood between the marginal maximum likelihood estimate of the recombination parameter *ρ* and the global maximum likelihood estimate (assuming a constant recombination rate), scaled by the ratio of the physical distance between the sites, *d_ij,_* to the total length of the sequence analyzed, *L*. Coalescent simulations (10,000) were carried out as described previously [19]. Because of the high level of recombination estimated for the African population, coalescent simulations were not feasible.

A parametric bootstrap was performed to obtain confidence intervals for the rate estimates for each population (Table 2). Simulations were carried out as above conditioning on the estimates of per-chromosome recombination rate, population mutation rate, and frequency spectrum of variation at each SNP for each population. The simulations were sorted for the top and bottom 97.5 percentiles.

### Estimates of LD and haplotype blocks

Standard LD estimates *r^2^* and *D′* were calculated for all pairs of sites, and significance was assessed by randomizing the positions of segregating sites 10,000 times [27]. Plots of LD decline with distance were constructed using DnaSP 4.0 [41]. Haplotype blocks were obtained using methods described [42]. We used two different LD classifications (*D′* and r^2^) for haplotype blocks and htSNP identification, and the major differences in numbers of blocks and htSNPs between populations remained the same regardless: *D′* is a measure of LD normalized by the largest LD value at the given allele frequencies, and r^2^ is the square of correlation coefficient of LD normalized by the variance in allele frequencies at the two loci. Haplotype blocks of various sizes were defined here as genomic regions with *D′* greater than or equal to 0.8 [42].

## Supporting Information

Figure S1 Worldwide Distribution of *P. falciparum* Isolates Used in this Study Parasites are color-coded as resistant to chloroquine (red), sensitive (black), or not tested (green).
(79 KB PDF).

Table S1 Putative Transporter Genes and Their Chromosomal Locations (29 KB DOC).

## Abbreviations

htSNP: haplotype tagging SNP
kb: kilobase
LD: linkage disequilibrium
PNG: Papua New Guinea
RJMCMC: Reversible Jump Markov Chain Monte Carlo
SE: Southeast
SNP: single nucleotide polymorphism
