# Supplementary material for: Recombination Hotspots and Population Structure in Plasmodium falciparum
Source: PLoS Biol. 2005 Sep 13;3(10):e335. doi: 10.1371/journal.pbio.0030335 (PMC1201364; doi:10.1371/journal.pbio.0030335)
Supplement: Supplementary file 1 [file pbio.0030335.sg001.pdf]

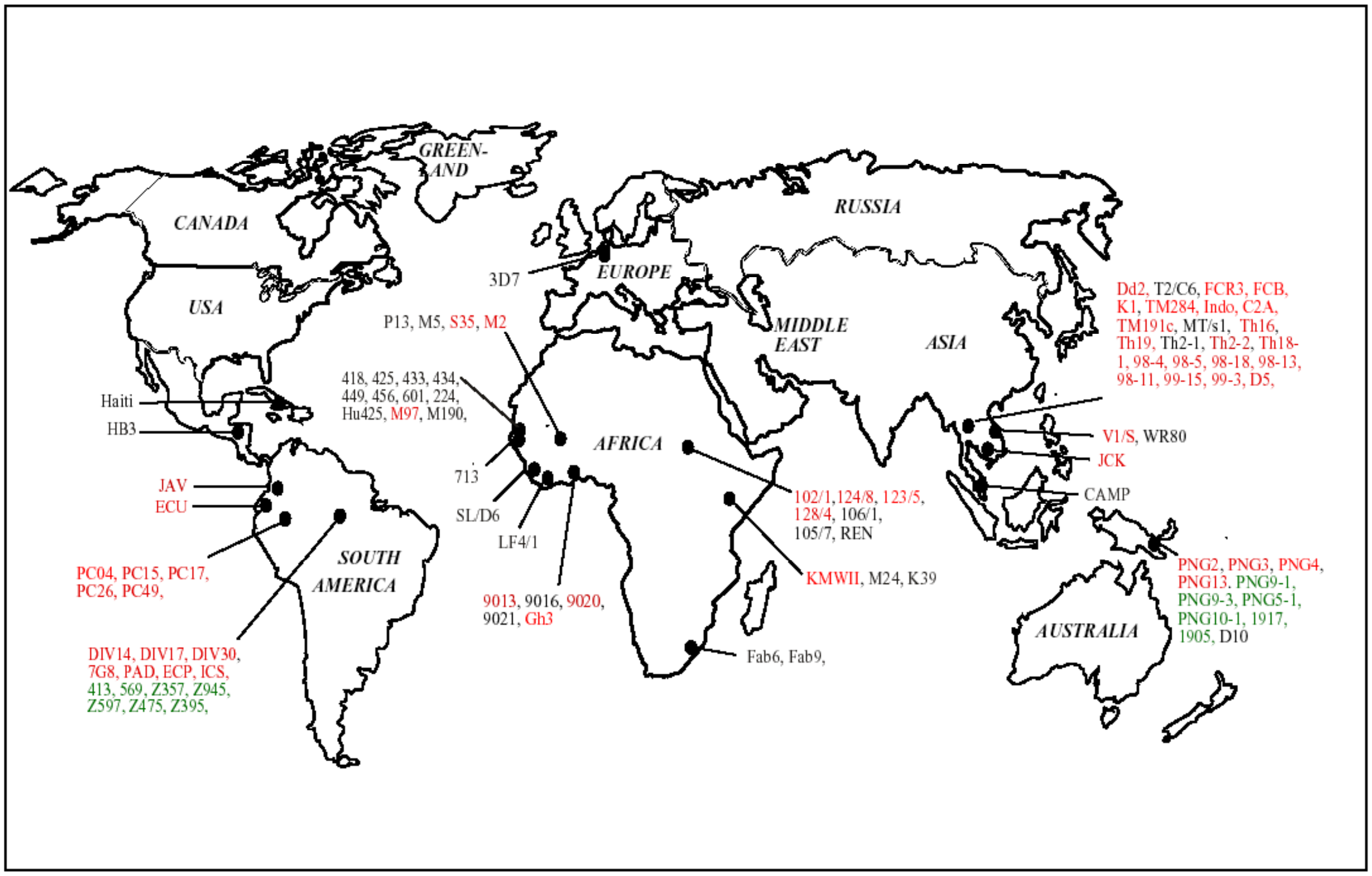
GREEN-
AND
CANADA
USA
RUSSIA
EUROPE
MIDDLE
EAST
ASIA
AFRICA
SOUTH
AMERICA
AUSTRALIA
3D7
P13, M5, S35, M2
418, 425, 433, 434,
449, 456, 601, 224,
Hu425, M97, M190,
713
SL/D6
LF4/1
9013, 9016, 9020,
9021, Gh3
Haiti
HB3
JAV
ECU
PC04, PC15, PC17,
PC26, PC49,
DIV14, DIV17, DIV30,
7G8, PAD, ECP, ICS,
413, 569, Z357, Z945,
Z597, Z475, Z395,
102/1,124/8, 123/5,
128/4, 106/1,
105/7, REN
KMWII, M24, K39
Fab6, Fab9,
Dd2, T2/C6, FCR3, FCB,
K1, TM284, Indo, C2A,
TM191c, MT/s1, Th16,
Th19, Th2-1, Th2-2, Th18-
1, 98-4, 98-5, 98-18, 98-13,
98-11, 99-15, 99-3, D5,
V1/S, WR80
JCK
CAMP
PNG2, PNG3, PNG4,
PNG13, PNG9-1,
PNG9-3, PNG5-1,
PNG10-1, 1917,
1905, D10
