# Supplementary material for: Recombination Hotspots and Population Structure in Plasmodium falciparum
Source: PLoS Biol. 2005 Sep 13;3(10):e335. doi: 10.1371/journal.pbio.0030335 (PMC1201364; doi:10.1371/journal.pbio.0030335)
Supplement: Supplementary file 2 [file pbio.0030335.st001.doc]

Supplementary Information is available on PLoS’s World-WideWeb site

([**http://biology.plosjournals.org/perlserv/?request=index-html&issn=1545-7885**](http://www.nature.com/) )

Supplementary Table 1. Putattive transporter genes and their chromosomal locations

Gene	Chr.	Locations on	PlasmoDB link	#SNPs	Putative function

Code	chr (bp)

G2	1	465876-471344	PFA0590w	3	ABCtransporter

G3	14	1953556-1956630	chr14.glm_514	4	ABC transporter(MDR2)

G4	3	138235-142332	PFC0125w	3	ABC transporter

G7	13	2072409-2075558	PF13_0271	3	ABC transporter

G8	11	1804269-1807036	PF11_0466	5	ABC transporter

G13	13	170076-175991	PF13_0019	2	NHE1

G14	12	602421-606757	PFL0685w	1	Multpile TM protein

G15	3	175096-177803	PFC0160w	1	Ankyrin repeat protein

G16	2	205889-207403	PFB0210c	3	Monosaccharide transporter

G18	2	480187-481432	PFB0535w	1	Multiple-TM protein

G20	3	818213-827472	PFC0875w	4	ATPase

G21	14	2649470-2654090	PF14_0622	2	Calcium_potassium channel

G22	12	1098119-1104058	PFL1315w	2	Potassium channel

G23	11	1268117-1268893	PF11_0338	1	Aquaporin

G24	5	990000-992054	PFE1185w	4	Cation transporter

G25	14	2911333-2914301	PF14_0679	2	Sulfate permease

G26	13	579728-585817	PF13_0079	3	Multiple TM + TPR

G29	2	528249-531923	PFB0580w	1	Multiple TM protein

G30	14	1229989-1232794	PF14_0292	1	GTPase

G33	14	1154308-1162647	PF14_0273	2	Multiple TM protein

G34	12	641545-643512	PFL0765w	2	Multuple TM MFS family

G41	9	667246-671716	PFI0785c	1	sugar transporter

G42	12	376547-387478	PFL0410w	4	ERLE domain protein

G43	14	3086131-3104141	PF14_0722	1	ERLE domain protein

G45	8	436476-443448	PF08_0048	1	Multpile TM protein

G46	13	1627342-1629511	MAL13P1.206	2	Phosphate transporter

G47	5	641135-644683	PFE0775c	3	Glycine transporter

G49	8	667662-671921	PF08_0078	3	ABC transporter

G52	10	1480517-1481422	PF10_0366	1	mt adenine transporter

G54	14	1100912-1103341	PF14_0260	4	Multpile TM protein

G55	14	536795-537838	PF14_0133	1	ABC transpoeter

G56	12	1192877-1199203	PFL1410c	4	ABC transporter

G60	12	1259609-1262077	PFL1480w	9	Sec13 othologt

G61	5	957885-962144	PFE1150w	5	MDR1

G62	7	307926-311020	MAL7P1.27	10	pfCRT

G69	12	528999-532792	PFL0590c	2	P-type- ATPase

G70	12	552096-553847	PFL0620c	1	Chroline transporter

These SNPs were initially collected for association study that was published previously (Mu, J. et al. Multiple transporters associated with malaria parasite responses to chloroquine and quinine*. Mol Microbi*o**l** 49, 977-989 (2003)).
